# Immediate and long-term relationship between severe maternal morbidity and health-related quality of life: a prospective double cohort comparison study

**DOI:** 10.1186/s12889-016-3524-9

**Published:** 2016-08-18

**Authors:** Mohd Noor Norhayati, Nik Hussain Nik Hazlina, Abd Aziz Aniza

**Affiliations:** 1 Department of Family Medicine, School of Medical Sciences, Health Campus, Universiti Sains Malaysia, 16150 Kota Bharu, Kelantan Malaysia; 2 Women’s Health Development Unit, School of Medical Sciences, Health Campus, Universiti Sains Malaysia, 16150 Kota Bharu, Kelantan Malaysia; 3 Faculty of Medicine, Universiti Sultan Zainal Abidin, 20400 Kuala Terengganu, Terengganu Malaysia

**Keywords:** Short Form-12 Health Survey, Quality of life, Severe maternal morbidity, Prospective double cohort

## Abstract

**Background:**

Given the growing interest in severe maternal morbidity (SMM), the need to assess its effects on quality of life is pressing. The objective of this study was to compare the quality of life scores between women with and without SMM at 1-month and 6-month postpartum in Kelantan, Malaysia.

**Methods:**

A prospective double cohort study design was applied at two tertiary referral hospitals over a 6-month period. The study population included all postpartum women who delivered in 2014. Postpartum women with and without SMM were selected as the exposed and non-exposed groups, respectively. For each exposed case identified, a non-exposed case with a similar mode of delivery was selected. The main outcome measures used were scores from the Short Form-12 Health Survey (SF-12).

**Results:**

The study measured 145 exposed and 187 non-exposed women. The group-time interaction of the repeated measure analysis of variance (RM ANOVA) showed no significant difference in the mean overall SF-12 physical component summary score changes (*P* = 0.534) between women with and without SMM. Similarly, the group-time interaction of the RM ANOVA showed no significant difference in the mean overall SF-12 mental component summary score changes (*P* = 0.674) between women with and without SMM. However, women with SMM scored significantly lower on a general health perceptions subscale at 1-month (*P* = 0.031), role limitations due to physical health subscale at 6-month (*P* = 0.019), vitality subscale at 1-month (*P* = 0.007) and 6-month (*P* = 0.008), and role limitations due to emotional problems subscales at 6-month (*P* = 0.008).

**Conclusions:**

Women with severe maternal morbidity demonstrated comparable quality of life during the 6-month postpartum period compared to women without severe maternal morbidity.

## Background

In the last several years, obstetric-outcome research has focused on maternal mortality and recently, more studies have addressed a wider range of hidden postpartum morbidity, such as backaches, abdominal pain and perineal pain. Findings have shown that these problems can persist over time and may affect the overall physical and mental health of women postpartum [1–3]. Quality of life is defined as an individual’s perception of his or her position in life in the context of the culture and value systems in which he or she lives and in relation to his or her goals, expectations, standards and concerns [4]. Worldwide, the findings on quality of life assessed using the Short Form-12 Health Survey (SF-12) reported better postpartum physical health compared to mental health. The mean physical component summary (PCS) scores from 48.5 to 51.4 at 5 to 6 weeks, 54.3 to 54.7 at 6 months, 54.1 to 55.0 at 12 months and 52.1 to 57.2 at 18 months postpartum. The mean mental component summary (MCS) scores reported were 29.2 to 53.3 at 5 to 6 weeks, 37.8 to 48.9 at 6 months, 40.8 to 49.9 at 12 months and 40.0 to 40.2 at 18 months postpartum [5–7].

The risk factors for quality of life have been identified in few studies. Most studies in the clinical research literature reported physical and mental health-related quality of life separately. In the former, better postpartum physical health was associated with better preconception health [5], better cardiovascular fitness [8] and more time elapsed since childbirth [5]. Vaginal delivery (compared to caesarean section) was shown to be significantly associated with physical health in a large sample of prospective cohorts [5] but not in a cross-sectional study [8]. Women with postnatal mood problems had poorer physical health [9], but women with prenatal mood problems did not show a similar association [5]. Da Costa et al. [8] reported that parity, body mass index, exercise participation, sleep quality and life stress were insignificant risk factors for physical health status. In addition, infant sex, social support, breastfeeding practice and infant colic showed no association [5]. In terms of mental health-related quality of life, better postpartum mental health was associated with better preconception mental health [5], multiparity [7], absence of prenatal and postnatal mood problems [5, 8, 9], having a female baby [5, 9], better sleep quality [8], less life stress [8], higher perceived social support [5, 8] and vaginal delivery [8].

Severe maternal morbidity refers to ‘potentially life-threatening conditions during pregnancy, childbirth or after termination of pregnancy from which maternal near miss cases would emerge’. Maternal near miss refers to ‘woman who nearly died but survived a complication that occurred during pregnancy, childbirth or within 42 days after termination of pregnancy’ [10, 11]. Based on Scottish confidential audit of severe maternal morbidity [12] focusing on postpartum haemorrhage and eclampsia, there were 13.8 severe acute maternal morbidity cases per 1000 deliveries in a tertiary hospital in Kelantan between November 2006 and October 2007 [13]. The effects of severe maternal morbidity on their general quality of life are a significant problem, as the women may be unprepared to deal with the complications. It has been shown that postpartum women suffer an array of symptoms and that a majority of them do not report such problems to their healthcare providers [14–16]. Limited study was conducted regarding the quality of life among postpartum mothers in Malaysia. Evidence regarding outcomes related to quality of life and severe maternal morbidity remains sparse, thus, meriting further investigation before any clear associations can be made. Therefore, this study is aimed to compare the quality of life scores between women with and without severe morbidity as measured by the SF-12 Health Survey at 1-month and 6-month postpartum recruited among those aged 18 and older and gave birth in tertiary hospitals in Kelantan during the year 2014. We hypothesized that the quality of life score is significantly lower in women with severe morbidity than those without severe morbidity at 1-month and 6-month postpartum.

## Methods

A prospective double cohort study design was applied. Women aged 18 and older who gave birth in the two tertiary referral hospitals in Kelantan during the year 2014 was followed-up for a 6-month period. The recruitment of participants occurred throughout the year 2014. Data collection for the 1-month follow-up was started in February 2014 and was completed in June 2015 for the 6-month cohort. There were two tertiary hospitals in Kelantan, which were situated in Kota Bharu, the capital city of Kelantan, had 66.0 % of hospitals beds and accounted for 54.0 % of total deliveries in the state. The exposure factor was the occurrence of severe maternal morbidity. The two cohorts of postpartum women with and without severe maternal morbidity exposure were compared and followed to determine the immediate and long-term consequences. The immediate and long-term postpartum periods [17, 18] were defined as 1-month and 6-month following delivery.

Postpartum women with and without severe maternal morbidity who fulfilled the inclusion and exclusion criteria were selected as the exposed and non-exposed groups, respectively. Identification of the exposed group was based on severe maternal morbidity criteria of four major conditions, including haemorrhagic disorders, hypertensive disorders, other systemic disorders and severe management indicators [10, 11]. For each exposed case identified, a non-exposed case with a similar mode of delivery (vaginal or caesarean delivery) was selected in the same facility. Women without severe maternal morbidity were considered as non-exposed group. To choose respondents of the non-exposed group, the study applied a computer-based simple random sampling to a predefined estimate of daily vaginal and caesarean deliveries. All birth outcomes including live birth, fetal death, neonatal death, singleton and multiple births were included. The study excluded women at less than 22 weeks of gestation, more than 42 days after the termination of pregnancy, with personal or family histories of diagnosed psychiatric disorders or who were not Malaysian citizens.

Sample size calculation was done by comparing two means using the Power and Sample Size Calculation software version 3.0.43 (Microsoft Corp., 2012). Taking the alpha of 0.05, power of 80 %, standard deviation of 4.2 [19] and detactable difference in the means of the quality of life scale of 1.5 based on clinical significance, the minimum required sample size was 124. However, after considering a non-response rate of 30 % in telephone-based survey [20, 21] and six months follow-up [22], the calculated sample size was 162 per group. Computer-based simple random sampling from a predefined estimate of daily vaginal and caesarean deliveries was applied for the non-exposed group.

During hospitalization, women with severe maternal morbidity (exposed) and women without severe maternal morbidity (non-exposed) were identified based on the study’s eligibility criteria. A research assistant trained in nursing reviewed the admission registers and medical records in delivery rooms and obstetrics and gynaecology wards daily. To decrease the risk of selection bias and to minimize the number of missed cases, all pregnancies and deliveries with any medical, not only potentially life-threatening conditions, were reviewed. The medical staffs were also asked regarding any cases that fulfilled the criteria. The researcher made the final choice for inclusion. They were then were briefed and invited into the study.

After signing the consent form, hospital- and home-based medical records were reviewed to retrieve patients’ information. The extracted information included the socio-demographic characteristics and obstetric history. The phone numbers were gathered from the study participants and at 1-month and 6-month (plus minus three days) following delivery, the participants were contacted to complete a structured telephone interview that incorporated the SF-12 Health Survey and the Medical Outcome Study (MOS) Social Support Survey. Similar method was applied in other studies [5, 23, 24]. For each follow-up, the research assistant made four or five attempts of phone calls before considering a participant is lost to follow-up. They were contacted either during the day or evening depending on their convenience.

The SF-36 is a well-known, generic, health-related quality of life questionnaire used worldwide [25], and the SF-12 is a shorter version that permits its application in large health studies. It measure eight domains of health and well-being under two overall scales, i.e., PCS and MCS. The scores vary from 0 to 100 for each scale with higher scores indicating better health status [26]. The International Quality of Life Assessment Project has prepared a true translation from United States (US) English into Malay of the SF-12v2® Health Surveys and tested with native speakers of Malay in Malaysia [27]. The Malay version of the SF-12v2® questionnaire was validated among 108 mothers who had had caesarean sections during a 1-month postpartum follow-up at the Obstetrics and Gynaecology Clinic in a tertiary teaching hospital. The internal consistency reliability for the SF-12 PCS and the SF-12 MCS were 0.749 and 0.701, respectively. Confirmatory factor solution showed that the two factor constructs, each with six items, had acceptable factor loadings, satisfactory absolutes and parsimonious fitness [Root mean square error of approximation (RMSEA) = 0.1, *x*^*2*^/*df* = 2.4] [28]. SF-12v2 was used to assess the mental and physical health of women in this study.

The MOS Social Support Survey measures perceived availability of functional social support [29] and has also been used in postpartum mothers [8, 30]. It rates items on a five-point scale ranging from 1 (none of the time) to 5 (all of the time), with higher scores indicating more support [29]. The Malay version of the questionnaire demonstrated acceptable factor loadings, domain-to-domain correlation and best fit [χ^*2*^(df) = 1.665 (61); *P*-value = 0.001; Tucker-Lewis index = 0.944; comparative fit index = 0.956; RMSEA = 0.068]. The internal consistency reliability of the four constructs ranged from 0.616 to 0.902 [31].

The data were entered and analysed using IBM SPSS Statistics version 22.0 (SPSS Inc., 2013). Independent *t* test and analysis of variance (ANOVA) were used for comparing means and chi-square test for comparing proportions between women with and without severe maternal morbidity. A repeated measure analysis of variance (RM ANOVA) was used to compare the outcome scores between women with and without severe maternal morbidity at 1-month and 6-month postpartum while controlling for the potential confounders based on statistical and clinical significance. The latter was chosen because they had been identified in previous research: mental health [9] was shown to be the potential confounder for physical health-related quality of life, and social support [5, 8] was the potential confounder for mental health-related quality of life. If a participant did not complete a particular question, their results will be excluded. However, all participants completed all questions.

## Results

Of 395 exposed women, 48 were excluded, as they were at less than 22 weeks of gestation (*n* = 32), were not Malaysian citizens (*n* = 10), were less than 18 years old (*n* = 3), had a personal history of diagnosed psychiatric disorder (*n* = 1) or a family history of psychiatric disorder (*n* = 3). Of the 347 eligible exposed women, 129 refused to participate and 55 agreed but were unable to be contacted (because nobody answered the telephone on more than three attempts or the telephone service had been disconnected) at 1-month postpartum. To match the number of eligible exposed women who could be recontacted, 347 non-exposed women were chosen. However, 104 of them refused to participate and four could not be contacted. Oversampling had been done earlier to address the possibility of unexpected dropouts and to achieve the calculated sample size. All of the remaining 163 exposed and 239 non-exposed women were interviewed at 1-month postpartum.

At 6-month postpartum, 145 exposed and 187 non-exposed women were interviewed and successfully provided complete information. The response rate for samples with completed data over the six months study period as compared with the number of all eligible exposed women was 41.8 % (145/347) and 53.9 % (187/347) of all non-exposed women. Therefore, to determine the changes in the quality of life scores, only data obtained from women who responded to both surveys were analyzed. That is why the final samples consisted of 145 exposed women and 187 non-exposed women.

Non-response bias analysis based on the sociodemographic profile was performed to assess the differences in characteristics between respondents and non-respondents [332 (47.8 %) vs 362 (52.2 %)] in order to justify the representativeness of the participants used for analysis. Age (*P* = 0.885), parity (*P* = 0.602), education level (*P* = 0.291), occupation (*P* = 0.671) and marital status (*P* = 0.359) did not differ significantly between the respondents and non-respondents. The respondents and non-respondents within each group of exposed and non-exposed women were also assessed. Within the exposed group, age (*P* = 0.726), parity (*P* = 0.564), education level (*P* = 0.869), occupation (*P* = 0.574) and marital status (*P* = 0.268) did not differ significantly between the respondents and non-respondents. Within the non-exposed group, age (*P* = 0.308), parity (*P* = 0.498), education level (*P* = 0.174), occupation (*P* = 0.135) and marital status (*P* = 0.539) were also did not differ significantly between the respondents and non-respondents.

Table 1 shows the findings of medical record review of sociodemographic characteristics and obstetric history of women with and without severe maternal morbidity in tertiary hospitals in Kota Bharu, Kelantan, Malaysia who gave birth in 2014. There were statistically significant differences in age, parity, duration of hospitalization and period of gestation between women with and without severe maternal morbidity. The baseline mean [standard deviation (SD)] of MOS social support score in women with [50.5 (10.98)] and without [55.2 (13.71)] severe maternal morbidity were statistically significant (*P* = 0.001). These variables were adjusted in subsequent analyses in order to determine the difference in physical and mental health quality of life at 1-month and 6-month postpartum between women with and without severe maternal morbidity.

**Table 1:** Sociodemographic characteristics and obstetric history of women with (*n* = 145) and without (*n* = 187) severe maternal morbidity

Variables	SMM (*n* = 145)				Non-SMM (*n* = 187)				*P* value
	mean	(SD^a^)	n	(%)	mean	(SD^a^)	n	(%)	
Sociodemographic characteristics									
Age (year)	31.6	(6.26)			29.2	(5.65)			<0.001^b^
Household income (RM/month)	2405.2	(1892.06)			2321.1	(2066.54)			0.547^b^
Education level									
Nil and Primary Secondary Tertiary			7 87 51	(4.8) (60.0) (35.2)			5 104 78	(2.7) (55.6) (41.7)	0.330^c^
Occupation									
Unemployed Self-employed Implementory Professional			74 8 41 22	(51.0) (5.5) (28.3) (15.2)			99 19 42 27	(52.9) (10.2) (22.5) (14.4)	0.340^c^
Marital status									
Married Single			145 0	(100.0) (0.0)			186 1	(99.5) (0.5)	0.378^c^
Past and present obstetric history									
Parity	3.1	(2.35)			2.7	(1.78)			0.043^b^
Hospital stay (day)	5.9	(3.79)			3.6	(2.23)			<0.001^b^
Booking									
Early (≤12 weeks) Late (> 12 weeks)			88 57	(60.7) (39.3)			114 73	(61.0) (39.0)	0.960^c^
Past pregnancy									
complications^d^ Absent Present			67 40	(62.6) (37.4)			92 37	(71.3) (28.7)	0.095^c^
Period of gestation									
Term (≥37 weeks) Preterm (<37 weeks)			110 35	(75.9) (24.1)			174 13	(93.0) (7.0)	<0.001^c^
Foetal outcome									
Live birth Stillbirth			143 2	(98.6) (1.4)			187 0	(100.0) (0.0)	0.107^c^

Abbreviations: *SMM* severe maternal morbidity, *RM* ringgit Malaysia, *ICU* intensive care unit

^a^Standard deviation

^b^Independent *t* test

^c^Chi-square test

^d^Past pregnancy complications refer to complications in a previous pregnancy. After excluding primiparous women, *n* = 107 for severe maternal morbidity group and *n* = 129 for non-severe maternal morbidity group

### Physical health-related quality of life and severe maternal morbidity

The PCS score distribution for women with severe maternal morbidity was normally distributed, ranging from 29.3 to 59.1 with a mean (SD) of 43.3 (5.30) at 1-month and ranging from 35.3 to 64.5 with a mean (SD) of 54.9 (3.55) at 6-month PCS score mean difference of 11.6 between the two follow-ups). The PCS score distribution for women without severe maternal morbidity was normally distributed, ranging from 27.0 to 57.6 with a mean (SD) of 43.7 (6.79) at 1-month and ranging from 38.9 to 63.0 with a mean (SD) of 55.3 (2.86) at 6-month (PCS score mean difference of 11.6 between the two follow-ups). ANOVA analysis showed no difference in PCS scores between women with and without severe morbidity at 1-month (*P* = 0.542) and 6-month (*P* = 0.182). However, women with severe maternal morbidity scored significantly lower on the general health perceptions subscale at 1-month (*P* = 0.031) and on the role limitations due to physical health subscale at 6-month (*P* = 0.019). Table 2 shows the SF-12 physical component and subscales description of quality of life.

**Table 2:** Comparison of SF-12 physical quality of life between women with and without severe maternal morbidity at 1-month and 6-month postpartum using ANOVA

	1-month					6-month				
SF-12	SMM (*n* = 145)		Non-SMM (*n* = 187)		*P* value^a^	SMM (*n* = 145)		Non-SMM (*n* = 187)		*P* value^a^
	mean	(SD^b^)	mean	(SD^b^)		mean	(SD^b^)	mean	(SD^b^)	
PF	42.5	(7.00)	43.7	(8.51)	0.164	56.0	(3.89)	56.6	(2.53)	0.096
RP	49.8	(6.31)	48.5	(6.98)	0.089	56.6	(2.29)	57.1	(1.74)	0.019
BP	48.3	(6.00)	48.2	(6.26)	0.873	54.9	(4.72)	55.0	(5.38)	0.884
GH	44.4	(8.38)	46.5	(8.80)	0.031	55.0	(5.11)	55.5	(4.66)	0.361
Overall PCS	43.3	(5.30)	43.7	(6.79)	0.542	54.9	(3.55)	55.3	(2.86)	0.182

Abbreviations: *SMM* severe maternal morbidity, *PF* physical functioning, *RP* role limitations due to physical health, *BP* bodily pain, *GH* general health perceptions, *PCS* physical component summary

^a^Analysis of variance

^b^Standard deviation

The group-time interaction of the RM ANOVA showed no difference in the PCS score changes (*P* = 0.534) between women with and without severe maternal morbidity (*n* = 332) after adjusting for age, parity, duration of hospital stay, period of gestation and mental health (Table 3). However, at both follow-ups, physical health as indicated by the mean PCS score was poorer for women with severe maternal morbidity than for women without severe maternal morbidity. At both times, the group effect of the RM ANOVA between women with and without severe maternal morbidity at both time levels showed no significant difference in the mean PCS scores [mean (95 % CI): 49.1 (48.53, 49.69) vs 49.5 (49.00, 50.02); *P* = 0.333].

**Table 3:** Comparison of physical quality of life between women with and without severe maternal morbidity at 1-month and 6-month postpartum using RM ANOVA

Groups	number	Desc Mean^a^ (SD^b^)			EMM^c^ (95 % CI^d^)			*F* stat^e^	*P* value^g^
		1-month	6-month	Diff.	1-month	6-month	Diff.	(*df* ^f^)	
SMM	145	43.3 (5.30)	54.9 (3.55)	11.6	43.2 (42.15, 44.21)	55.0 (54.49, 55.59)	11.8	0.388 (1, 325)	0.534
Non-SMM	187	43.7 (6.79)	55.3 (2.86)	11.6	43.8 (42.94, 44.73)	55.2 (54.71, 55.66)	11.4		

Abbreviation: *SMM* severe maternal morbidity

^a^ Descriptive mean

^b^Standard deviation

^c^Estimated marginal mean

^d^Confidence interval

^e^F statistic

^f^Degree of freedom

^g^Group-time interaction of repeated measure analysis of variance (Model assumptions met). Adjusted for age, parity, duration of hospital stay, period of gestation and mental health

Residual plots indicate that overall model fitness and equal variance assumption were satisfied. Normality of standardized residuals was appropriate. The variable functional forms for the age, parity, duration of hospital stay and MCS score were appropriate. There were seven outliers when plotting standardized residuals against the predicted value. However, the changes in *F* statistic (0.154) did not change the significance level (*P* = 0.695) after removal of these outliers. Therefore, the outliers were retained in the model and the above model was considered to be the final model.

### Mental health-related quality of life and severe maternal morbidity

The MCS score distribution for women with severe maternal morbidity was normally distributed, ranging from 36.7 to 69.1 with a mean (SD) of 56.1 (5.43) at 1-month and ranging from 50.2 to 64.5 with a mean (SD) of 57.8 (2.13) at 6-month (MCS score mean difference of 1.7 between the two follow-ups). The MCS score distribution for women without severe maternal morbidity was normally distributed, ranging from 34.1 to 70.5 with a mean (SD) of 56.8 (6.39) at 1-month and ranging from 40.8 to 65.1 with a mean (SD) of 58.1 (2.67) at 6-month (MCS score mean difference of 1.3 between the two follow-ups). The ANOVA analysis showed no difference in the MCS scores between women with and without severe maternal morbidity at 1-month (*P* = 0.280) and 6-month (*P* = 0.270). However, at 1-month, women with severe maternal morbidity scored significantly lower on the vitality subscale (*P* = 0.007) and at 6-month, on both the vitality (*P* = 0.008) and the role limitations due to emotional problems (*P* = 0.008) subscales. Table 4 shows the SF-12 mental component and subscales description of quality of life.

**Table 4:** Comparison of SF-12 mental quality of life between women with and without severe maternal morbidity at 1-month and 6-month postpartum using ANOVA

	1-month					6-month				
SF-12	SMM (*n* = 145)		Non-SMM (*n* = 187)		*P* value^a^	SMM (*n* = 145)		Non-SMM (*n* = 187)		*P* value^a^
	mean	(SD^b^)	mean	(SD^b^)		mean	(SD^b^)	mean	(SD^b^)	
VT	52.5	(6.28)	54.4	(6.78)	0.007	58.2	(5.32)	59.6	(4.79)	0.008
SF	50.5	(6.17)	50.9	(6.87)	0.596	56.6	(2.20)	56.1	(4.95)	0.281
RE	51.9	(6.92)	51.4	(7.88)	0.554	55.8	(1.56)	56.2	(0.86)	0.008
MH	55.3	(5.53)	56.1	(5.91)	0.231	58.8	(2.81)	58.9	(3.47)	0.806
Overall MCS	56.1	(5.43)	56.8	(6.39)	0.280	57.8	(2.13)	58.1	(2.67)	0.270

Abbreviations: *SMM* severe maternal morbidity, *VT* vitality, *SF* social functioning, *RE* role limitations due to emotional problems, *MH* mental health, *MCS* mental component summary

^a^Analysis of variance

^b^Standard deviation

The group-time interaction of the RM ANOVA showed no difference in the MCS score changes (*P* = 0.674) between women with and without severe maternal morbidity (*n* = 332) after adjusting for age, parity, duration of hospital stay, period of gestation and social support (Table 5). However, at both follow-ups, the mental health as indicated by the mean MCS score was poorer for women with severe maternal morbidity than for women without severe maternal morbidity. At both times, the group effect of RM ANOVA between women with and without severe maternal morbidity showed no significant difference in the mean MCS scores [mean (95 % CI): 57.1 (56.46, 57.64) vs 57.4 (56.89, 57.92); *P* = 0.406].

**Table 5:** Comparison of mental quality of life between women with and without severe maternal morbidity at 1-month and 6-month postpartum using RM ANOVA

Groups	number	Desc Mean^a^ (SD^b^)			EMM^c^ (95 % CI^d^)			*F* stat^e^ (*df* ^f^)	*P* value^g^
		1-month	6-month	Diff.	1-month	6-month	Diff.		
SMM	145	56.1 (5.43)	57.8 (2.13)	1.7	56.2(55.20, 57.29)	57.9 (57.43, 58.29)	1.7	0.177 (1, 325)	0.674
Non-SMM	187	56.8 (6.39)	58.1 (2.67)	1.3	56.8 (55.85, 57.66)	58.0 (57.67, 58.42)	1.2		

Abbreviation: *SMM* severe maternal morbidity

^a^Descriptive mean

^b^Standard deviation

^c^Estimated marginal mean

^d^Confidence interval

^e^ *F* statistic

^f^Degree of freedom

^g^Group-time interaction of repeated measure analysis of variance (Model assumptions met). Adjusted for age, parity, duration of hospital stay, period of gestation and social support

Residual plots indicate that overall model fitness and equal variance assumption were satisfied. Normality of standardized residuals was appropriate. The variable functional forms for the age, parity, duration of hospital stay and MOS social support score were appropriate. There were six outliers when plotting standardized residuals against the predicted value. However, the changes in *F* statistic (0.003) did not change the significance level (*P* = 0.959) after removal of these outliers. Therefore, the outliers were retained in the model and the above model was considered to be the final model.

## Discussion

In the present study, the definition of and criteria for severe maternal morbidity recommended by the World Health Organization [11] were used to evaluate its effects on physical and mental health-related quality of life for the first time. Therefore, no previous studies were available for comparison. Results show that the overall physical and mental health-related quality of life of women with severe maternal morbidity seemed not to differ from that of women without severe maternal morbidity. Nonetheless, study results showed that women who experienced severe maternal morbidity demonstrated poorer physical and mental health scores in four of the eight subscales of the SF-12, including general health perceptions (1-month), vitality (1- and 6-month) and role limitations due to both physical and emotional problems (6-month).

### Physical health-related quality of life

Results of the present study show that the overall physical health of women with severe maternal morbidity did not differ from that of women without severe maternal morbidity over the six months postpartum period. Nonetheless, the physical health subscales demonstrated that women with severe maternal morbidity have poorer general health perceptions and experienced greater limitations due to physical difficulties. In which, at one month, these women had significantly lower scores on the subscale for general health perceptions, and at six months, on the subscale for role limitations due to physical health. The subscales for general health perceptions and role limitations due to physical health reported a 2.1 and 0.4 point difference, respectively, between those with severe maternal morbidity and those without. In addition to being statistically significant, a difference of 2 to 3 points is believed to be clinically significant [32]. Therefore, the present study can conclude that severe maternal morbidity has significant effects, both statistically and clinically, on the general health perceptions of women.

Most of the studies in the literature looked at the individual signs and symptoms that developed in the postpartum period, making comparison difficult. Fatigue, physical exhaustion, sleep-related problems and pain are common physical problems in the postpartum period [33]. Nagpal et al. [34], using the Mother Generated Index on 197 women at five weeks postpartum, reported that back pain, tiredness and inability to do routine duties were more common among mothers with caesarean sections. Women who underwent caesarean sections had worse physical health than women who gave birth vaginally, because they were recovering from childbirth and surgery and needed to attend to infant care responsibilities [1, 5]. However, physical morbidities were not necessarily related to the method of birth [35]. In a sample of 1323 women in the United States, approximately 80 % of whom had delivered vaginally, more than two-thirds (69 %) experienced at least one physical health problem, such as backache and abdominal pain, following childbirth. The presence of physical health problems consistently correlated with functional limitations, such as child care and daily activities [3].

Similar findings were reported by Cheng and Li [33]. In their study, one-third of women experienced body aches, tiredness and breast problems during the first month and bowel problems between 1 and 3 months postpartum. Most of these symptoms decreased between 3 and 6 months, except for backache and tiredness. After six months postpartum, they were more likely to experienced headaches, sexual dysfunctions and sleep disorders. Women may regard physical symptoms, such as tiredness and backaches, as natural physical postpartum changes, and thus may not seek medical attention [33].

Apart from the subjective physical problems, the differences in physical health can probably be explained by the added responsibilities for infant care and lack of preparation for and knowledge about postpartum. These factors have been documented as sources of fatigue for mothers [36]. In a randomized control trial, healthy women attending antenatal classes that included education on changes during pregnancy, exercises during pregnancy, stages of labour, proper positioning during labour and postpartum care showed better quality of life from 6 to 8 weeks to one year postpartum. Knowledge about the changes during pregnancy and the postpartum period contributed to the highest score for the quality of life domains [37]. Lack of preparation for the postpartum experience was associated with more physical and mental ill health among women who had had uncomplicated deliveries. Women who were less prepared were less likely to trust their care providers and more likely to regard their health as poor [38]. Given the increased responsibilities and lack of postpartum preparedness, women facing unexpected, severe obstetric complications, as did those in the present study, might well experience or perceive similar, if not worse, consequences.

Results of the present study show that at one month postpartum, the mean overall SF-12 PCS scores for both groups were exceptionally low (approximately 43) and below the average norm [39]. Other studies in developed countries have reported mean SF-12 PCS scores of approximately 50 between 5 and 6 weeks postpartum [5, 6]. Although the present study was not intended to compare the differences among countries or cultures, several factors may come into play. A study comparing the quality of life between mothers in Austria and Japan showed that women with higher standards of living, better incomes and better social support from family, friends and important others had better physical quality of life [40]. Despite this, subsequent evaluations of the mean overall SF-12 PCS scores in the present study showed that they were much higher at six months than at one month, suggesting that, in general, the women were more satisfied with their physical health as time passed.

### Mental health-related quality of life

Results of the present study showed that the overall mental health of women with severe maternal morbidity did not differ from that of women without severe maternal morbidity over the six months postpartum period. Women with severe maternal morbidity scored significantly lower on the vitality subscale at one month and six months, and on the subscale for role limitations due to emotional problems at six months. The vitality subscales reported a 1.4 to 1.9 point difference, while the role limitations due to emotional problems subscale reported a 0.4 point difference. Although the vitality subscale results are significantly lower for women with severe maternal morbidity, they are not considered clinically significant [32].

A study that assessed women with high postpartum morbidity based on the presence of 13 physical symptoms showed that they had six times more poor emotional health than women without morbidity. The emotional health was self-rated by a single item as poor (fair or poor) or good (excellent, very good, or good) [3]. Psychological health problems, described as a low mood, a depressive mood or crying with no reason, were reported by 84.4 % of women between 2 and 6 months postpartum [35]. However, neither of these studies investigated the association between severe maternal morbidity and quality of life.

Unexpectedly and in contrast to other studies, the present study observed better mental health-related quality of life. Using the same questionnaire and approximately similar time intervals, the present study demonstrated higher mean overall SF-12 MCS scores among women with severe maternal morbidity than among women in the general populations of the United Kingdom and the United States [5, 6]. The present study’s mean overall SF-12 MCS scores, which ranged from 56 to 58, were above the average norm [39], while the United Kingdom and the United States studies reported ranges from approximately 37 to 50. The minimum difference of six points indicates a moderate-to-large effect [39] on maternal mental health, which may be attributed to coping mechanisms. A metasynthesis of qualitative studies showed that women who anchored themselves in religious beliefs, placed more value on their families and displaced their emotions to their child had better internal ability to cope with difficulties [41]. The direction and magnitude of effect of the mean overall SF-12 MCS scores from 1 to 6 months was small and translated to a slight improvement in postpartum mental health.

The explanation for the smaller difference in the mental health compared to physical health during the same period of time could be explained by the qualitative counterpart that was reported elsewhere. Since the early postpartum period, these women had expressed their acceptance regarding the severe morbid conditions that has occurred around the time of childbirth. It was anchored to religious faith in the form of contentment and reliance on God. This has formed an excellent coping mechanism for the recovery of the women. Therefore, mental health quality of life that has shown better outcome at 1-month did not show much increment at 6-month postpartum. The physical health quality of life, naturally, showed improvement as time passed.

This study is unique, as it illustrates the effects of severe maternal morbidity on physical and mental health until 6 months postpartum. The few studies published in recent years have focused only on the patterns and factors associated with severe maternal morbidity. A limited number of international studies have evaluated the consequences of severe maternal morbidity. At the national level, this study is the first, thus providing a paramount contribution to the current knowledge on maternal health. The study employed a prospective design and a well-validated questionnaire and controlled for important confounding factors previously identified in the literature. With regard to clinical implications, the present study has increased the understanding of postpartum health of women with severe maternal morbidity.

This study is not without limitations. Although the women were followed up in the community, they were recruited from the hospitals. Therefore, the present study was not a population-based study, and its results cannot be generalized to the Kelantanese population. However, the study may be representative of women who have access to the healthcare system and delivered in tertiary hospitals. Postpartum stage at evaluation seems to be important. Studies conducted at an earlier postpartum period, within the first two weeks, showed impaired quality of life with the SF-12 [38] and the SF-36 [42] among women in the general population. Results of the present study might have differed if it had assessed quality of life at an earlier period following delivery. However, evaluation at one month postpartum was justified in view of the aim to compare the women’s quality of life difference between early to later postpartum period.

Recruitment of women to this cohort study was carefully planned to obtain an equal proportion of women who had experienced each of the two modes of delivery among both the exposed and non-exposed groups. However, due to the non-response rate, 80.7 % of women with severe maternal morbidity and 67.9 % of women without severe maternal morbidity had delivered via caesarean section. However, this disproportion in the mode of delivery does not have profound effects on the findings. If all the physical and mental health outcome findings had been significant, it would be possible that a mode of delivery effect was present.

### Recommendations

Postpartum experiences of women may be minimized by setting appropriate expectations especially in the presence of unfavourable consequences of pregnancy and delivery. This can be achieved by childbirth preparation and education classes, which have been shown to improve physical functions and trust in healthcare providers and to reduce physical and depressive symptoms [38].

When medical assistance from healthcare providers is not sought, unresolved physical and mental health conditions may affect maternal health. Therefore, postnatal home visit personnel should actively screen postpartum mothers for physical problems and help them manage those conditions appropriately. In addition to birthing plans, postnatal support plans are important. Healthcare providers can assist mothers in enlisting a source of support for managing routine household activities and infant care responsibilities. Social support from family, friends and significant others is important to better physical and physiological quality of life [40]. This study observed poorer physical health but better mental health in comparison to populations in developed countries. Further linear regression analysis must be performed to determine the strength of the relationship between physical and mental health in this study’s postpartum population.

The present study suggests three priorities for future research. First, replicate studies in other populations would produce a more comprehensive national and international picture of the effects of severe maternal morbidity. Second, cross-cultural exploration would explain the differences in quality of life caused by different living conditions. Finally, listening to the voices of women who have experienced very severe maternal morbidity or maternal near miss may further explain and add meaning to the results.

## Conclusions

In general, women with and without severe maternal morbidity demonstrate comparable overall quality of life during the immediate and the long-term postpartum periods. Nonetheless, women with severe maternal morbidity have poorer quality of life in the dimensions of general health perceptions, vitality and limitations due to physical and mental problems that affect their roles than do women without severe maternal morbidity.

## Abbreviations

BP: Bodily pain
CI: Confidence interval
EMM: Estimated marginal mean
GH: General health perceptions
MCS: Mental component summary
MH: Mental health
MOS: Medical outcome study
PCS: Physical component summary
PF: Physical functioning
RE: Role limitations due to emotional problems
RM: Ringgit Malaysia
RM ANOVA: Repeated measure analysis of variance
RMSEA: Root mean square error of approximation
RP: Role limitations due to physical health
SD: Standard deviation
SF: Social functioning
SF-12: Short Form-12 Health Survey
SF-36: Short Form-36 Health Survey
SMM: Severe maternal morbidity
VT: Vitality
